# Toward High-Throughput Virtual Screening via Quasi-Adiabatic Quantum Geometry Optimization Workflow

**DOI:** 10.1021/acs.jctc.6c00751

**Published:** 2026-08-27

**Authors:** Leonardo U. Masci, Fabio Tarocco, Domenico Bonanni, Matteo Barbieri, Simone Rizzo, Andrea R. Beccari, Leonardo Guidoni

**Affiliations:** † 18798 Exscalate, Dompé Farmaceutici S.p.A., 80131 Naples, Italy; ‡ Department of Information Engineering, Computer Science and Mathematics, 9303 University of L’Aquila, 67100 L’Aquila, Italy; § Department of Physical and Chemical Sciences, University of L’Aquila, 67100 L’Aquila, Italy; ∥ Data Science and Computation Facility, Istituto Italiano di Tecnologia, 16163 Genoa, Italy; ⊥ E4 Computer Engineering SpA, 42019 Scandiano, Italy

## Abstract

This work addresses the challenge of molecular geometry optimization at post-Hartree–Fock levels of theory using quantum computing methodologies. We present a novel potentially quasi-adiabatic approach based on the Variational Quantum Eigensolver (VQE) algorithm for efficient exploration of potential energy surfaces. The method combines quantum-circuit emulators with classical optimization routines and uses the Hellmann–Feynman theorem, including Pulay corrections, together with a finite-difference scheme to compute forces. Our implementation uses GPU-accelerated quantum-circuit operations within a parallelized framework, achieving computational efficiency by warm-starting the wavefunction and reoptimizing it at each subsequent geometry-optimization step. Benchmark results on small molecular systems demonstrate the feasibility of this hybrid quantum-classical approach, with detailed comparisons against established quantum chemistry methods, including CASCI and DFT optimizations. We discuss the current limitations, computational advantages, and potential future developments of this methodology in the context of quantum chemistry for drug discovery applications.

## Graphical abstract

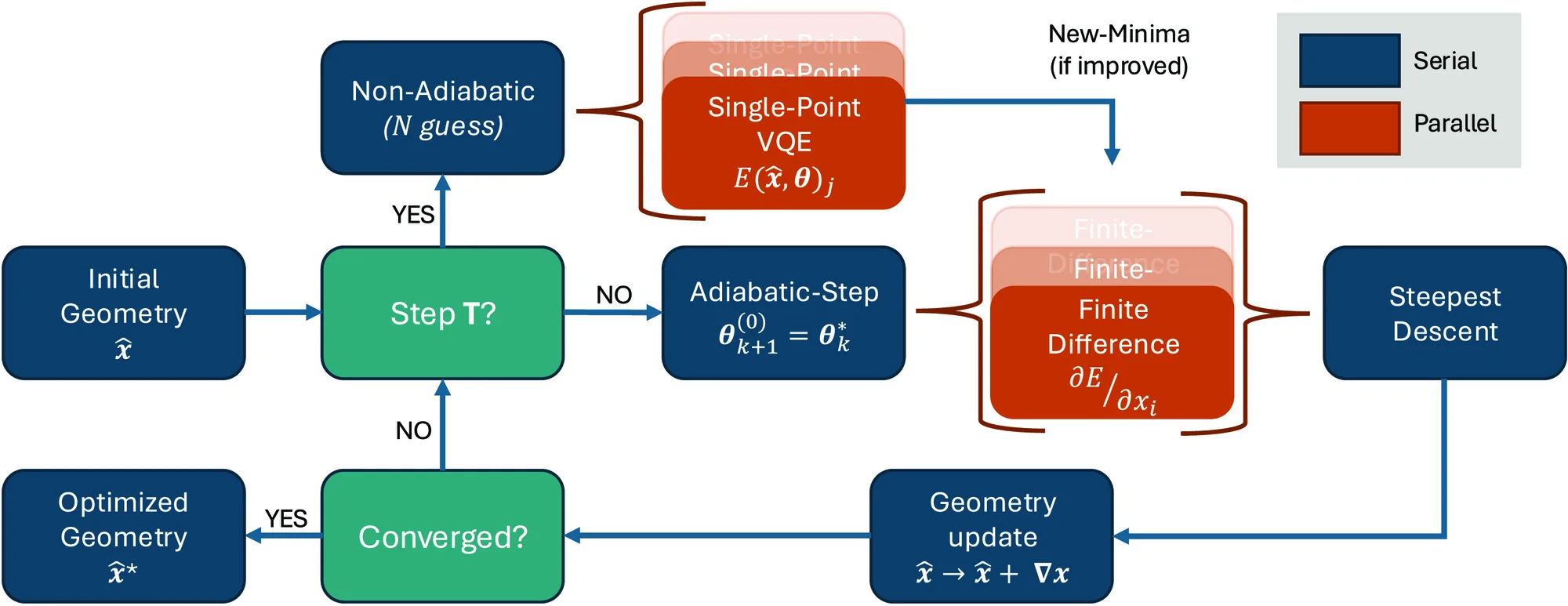

## 1 Introduction

In computer-aided drug discovery (CADD),^1^ high-throughput virtual screening (HTVS)^2^ is a computational technique aiming at filtering huge databases of chemical compounds to identify a small subset of promising candidates (hits/leads) to bind a specific biological macromolecular target, usually a protein, an enzyme, or even a DNA fragment.^3^

By predicting the most energetically favorable conformation of a ligand to its target macromolecule, HTVS acts as a high-speed, cost-effective filter, replacing physical screening to predict binding affinity, thus reducing the number of molecules that undergo costly lab synthesis and testing and, therefore, saving time and resources.

The first step of a virtual screening (VS) workflow is the generation of the three-dimensional structure of a candidate molecule. Usually, this step is carried out by starting from a lightweight Simplified Molecular Input Line Entry System (SMILES)^4^ file, which is a standard, compact, text-based format for storing chemical structures in databases. In the SMILES format, a molecular structure is encoded in a linear string of characters, making it an efficient method for storage and retrieval in large-scale HTVS campaigns. From a SMILES string, energetically stable three-dimensional structures (conformers) of a candidate molecule can be generated by searching for the molecular conformations that locally minimize the PES, a model of intramolecular interactions using physics-based functional forms and ad-hoc parametrized biomolecular force fields.^5^ Finally, such three-dimensional structures are used as input for fast scoring functions (SFs)^6^ that estimate the binding affinity between the candidate molecule and the target at hand.

However, despite its computational efficiency, some chemical details are omitted in the input structures generated using the above-mentioned workflow. Furthermore, being recovered from a classical PES, the energy and structure of the output molecule do not take into account quantum nuclear effects (e.g., zero level energies), particularly important in molecules with numerous hydrogen atoms, quite common among VS chemical libraries. Using more accurate molecular structures in input for the SF used in VS campaigns would allow more reliable binding affinity estimates, which in turn would reduce expensive in vitro tests of inactive molecules identified in silico as promising (false positives) and the discarding of promising compounds not identified as active in VS campaigns (false negatives).

Molecular geometry optimization represents a fundamental challenge in computational quantum chemistry, particularly when seeking high accuracy through post-Hartree–Fock (post-HF) methods.^7–9^ The optimization of molecular structures at post-HF levels of theory requires repeated evaluation of electronic energies and nuclear gradients,^10^ making it computationally demanding even for moderately sized systems. Current state-of-the-art approaches typically rely on density functional theory (DFT)^11^ for routine applications or expensive coupled-cluster^12^ methods when higher accuracy is required. The emergence of quantum computing offers promising alternatives for facing these challenges, with the Variational Quantum Eigensolver (VQE)^13^ serving as a popular near-term quantum algorithm for quantum chemistry applications.^7,14–19^

In this work, we introduce a potentially[Fn fn1] quasi-adiabatic VQE-based approach for molecular geometry optimization. Our methodology combines quantum circuit simulations for electronic structure calculations with classical minimization algorithms for geometry optimization, in a similar fashion as already implemented approaches.^20–24^ We implement force calculations through the Hellmann–Feynman theorem^25^ with appropriate corrections for basis set incompleteness,^10^ due to the dependence of the basis functions on nuclear coordinates, exploiting the well-known finite-difference approach for implementation simplicity. The practical implementation can also leverage GPU-accelerated quantum simulators to enable efficient exploration of potential energy surfaces.

Given the intrinsic limitations of current and near-future NISQ hardware and emulation software, we focus on Dompé Keys molecular fragments, whose size aligns with the small chemical systems typically explored in recent quantum chemistry studies,^20,22,26^ such as benchmarking efforts on VQE for molecules within similar qubit ranges. Dompé Keys comprehensively describe the chemical library used as input for the SF adopted by LiGen,^2^ VS software used by the EXSCALATE[Fn fn2] Platform in executing VS campaigns.

Our contributions include: (i) development of a hybrid quantum-classical framework for geometry optimization at post-HF levels, (ii) implementing efficient, VQE-compatible force-evaluation schemes designed for parallel execution, (iii) benchmarking against established quantum chemistry methods on representative molecular systems, and (iv) comprehensive analysis of computational performance and accuracy trade-offs. Through systematic evaluation on test systems, we demonstrate the feasibility of this approach and identify key areas for future improvement.

The remainder of the manuscript is organized as follows: Section 2 introduces the theoretical background, covering VQE, post-HF methods, and force calculations. Section 3 reviews recent related studies in the literature. Section 4 details our methodological approach, while Section 5 presents the overall computational framework. Section 6 reports the computational setupdiscusses and the molecular system studied. Section 7 collects the results and benchmarks. Section 8 offers a discussion and concludes with perspectives for future developments.

## 2 Theory and Background

In this section, we go through the theoretical background that is required to smoothly read the development part of the manuscript.

### 2.1 Quantum Computing for Quantum Chemistry

One of the fields in which quantum computing has found most application is, above all, quantum chemistry.^27^ The use of quantum computing to solve quantum chemistry problems is motivated not only by the exponential scaling of classical algorithms for simulating exactly quantum systems but also by the intrinsic limitations of classical optimization methods. Moreover, classical electronic-structure methods often struggle when dealing with strongly correlated electrons, such as those found in systems involving transition-metal complexes, metalloproteins, metal-containing ligands, or radical species. Quantum computers, on the other hand, can naturally represent and manipulate highly entangled quantum states, making them promising platforms for accurately treating these challenging systems.

Second quantization provides the natural framework for this mapping, expressing molecular Hamiltonians in terms of creation and annihilation operators. This formalism facilitates the translation of quantum chemistry problems into the language of quantum computing, where quantum gates manipulate qubit states to simulate electronic wavefunctions. Given a molecule and a set of spin molecular orbitals (MOs), the second-quantized electronic Hamiltonian is defined as follows

1 $$Ĥ=\sum_{\mathrm{ij}}h_{\mathrm{ij}}{â}_{i}^{†}{â}_{j}+\frac{1}{2}\sum_{\mathrm{ijkl}}\Gamma_{\mathrm{ijkl}}{â}_{i}^{†}{â}_{j}^{†}{â}_{k}{â}_{l}$$

where *a*_*i*_^†^ and *a*_*i*_ are creation and annihilation operators, respectively, while *h*_*ij*_ and Γ_*ijkl*_ are the one-body and two-body integrals. In the summation, the indices of eq 1 iterate over the set of spin MOs that can be composed of Hartee–Fock orbitals. or the desired molecular basis. To convert from Fermionic space to qubit space is necessary to apply a Fermion-to-qubit mapping. Different methods exist to convert Fermionic operators to the bosonic space of the qubits, above all the most popular are Jordan–Wigner,^28^ Parity,^29^ Bravy–Kitaev,^30^ but also custom and ad-hoc mappings can be defined.^31,32^ Here, we proceed the explanation by exploiting the Jordan–Wigner mapping. The Jordan–Wigner mapping allows for a more intuitive and straightforward transformation, maintaining an almost 1-to-1 correspondence between Slater determinants and states in the computational basis, so in the qubit space. Each MOs is going to be mapped to one qubit, and the encoding represents the occupation of that specific spin orbital, as for Slater Determinants. In particular, the *i*-th qubit will be associated with the *i*-th spin orbital and, in order to modify and act on the specific orbital, the associated *i*-th Fermionic operator will be translated as

2 $$a_{i}^{†}=Q_{i}^{†}\prod_{j=0}^{i-1}Z_{j}\;\mathrm{an}d\;a_{i}=Q_{i}\prod_{j=0}^{i-1}Z_{j}$$

where $Q_{i}^{†}=\frac{1}{2}\left(X_{i}-{\mathrm{iY}}_{i}\right)$ , $Q_{i}=\frac{1}{2}\left(X_{i}+{\mathrm{iY}}_{i}\right)$ , and *i* iterates over the set of MOs. They can be associated with creation and annihilation operators in the qubit space, respectively. As well as the Fermionic creation and annihilation operators act to modify the occupancy of the relative spin-MO, the qubit operators act to change the state of the qubit related to the specific spin-MO. The full correspondence is finalized with a series of Pauli-Z matrices that allow computing the parity of the state and accounting for the Fermionic anticommutation between *a* and *a* ^†^. Using eqs 2 in [Disp-formula eq1], the new Hamiltonian defined in the qubit space, **Ĥ**, can be written as

3 $$Ĥ=\sum_{i}^{L}c_{i}{\mathrm{P̂}}_{i}=\sum_{i}^{L}c_{i}\overset{N_{\mathrm{MO}}}{\underset{j=0}{⊗}}{\mathrm{\sigma ̂}}_{j}^{i}$$

where each σ̂_*j*_ ∈{ *X, Y, Z, I*} is a Pauli matrix acting on the *j*-th qubit, *c*_*i*_ is a coefficient relative to the *i*-th Pauli string *P̂*_*i*_, *N*_*MO*_ *is the number of qubit or number of spin-MOs, and L, proportional to N*_*MO*_^4^, *is the number of Pauli strings composing the qubit Hamiltonian.*

### 2.2 Variational Quantum Eigensolver

The Variational Quantum Eigensolver is a hybrid quantum-classical algorithm designed for near-term quantum devices.^13^ Since about a decade, the method has been largely studied (and criticized) for its employment in near-term applications designed for quantum chemistry.^13–19^ The VQE is generally used in place of a classical CI solver a-la CASCI for implementing hybrid quantum-classical pipelines for which, ideally, the resolution of the classical part is unfeasible (An exhaustive review on the VQE method can be found in the references.^19^) The VQE algorithm bases its resolution on the *Rayleigh-Ritz Variational Principle*.^33^ The principle states that given Hamiltonian *H* and the associated ground-state energy *E*_0_, for any trial wavefunction |ψ⟩, the energy obtained by the trial state

4 $$E_{0}\leq \left\langle \Psi \right|H\left|\Psi \right\rangle$$

Therefore, the energy of any wavefunction is strictly lower bounded by the ground state energy *E*_0_ of the system associated with *H*. The VQE method, exploiting the principle explained in eq 4, is meant to variationally optimize a parametrized wavefunction until it reaches, ideally, the optimal wavefunction i.e., the best approximation to the ground state. The trial wavefunction is a *Parametrized Quantum Circuit (PQC)*, denoted with |ψ­(θ)⟩, where θ is the set of parameters that are optimized by the VQE procedure. If we now substitute the newly defined PQC in eq 4, we obtain

5 $$E_{0}\leq E\left(\theta \right)=\frac{\left\langle \psi \left(\theta \right)\right|H\left|\psi \left(\theta \right)\right\rangle }{\left\langle \psi \left(\theta \right)\right|\psi \left(\theta \right)\rangle }$$

Going into the detail of eq 5, without loss of generality, we can define a PQC as

6 |ψ(θ)⟩=U(θ)|0⟩

i.e., a parametrized unitary evolution applied to the qubit-vacuum state, or in general, to a reference state (for quantum chemistry applications, the starting guess is always the HF state). The parameter vector **θ** is composed by a set of *j* real-valued parameters, where *j* is associated with the number of parametrized unitaries *U*(θ_*j*_) that are used to compose the full *U*(**θ**). In particular, the PQC can be written as

7 $$U\left(\theta \right)=\prod_{j=N-1}^{0}U\left(\theta_{j}\right)$$

After introducing the ideal form of the trial wavefunction, the VQE algorithm can be defined as the task of finding the set of parameters **θ** ^★^ that minimizes the expectation value of a given operator, *H*, by iteratively improving the wavefunction, in order to find the best approximation of the ground state *E*_0_, i.e.

8 $$E_{0}∼E\left(\theta^{★}\right)=\min_{\theta \in R^{j}\;}\frac{\left\langle \psi \left(\theta \right)\right|H\left|\psi \left(\theta \right)\right\rangle }{\left\langle \psi \left(\theta \right)\right|\psi \left(\theta \right)\rangle }$$

If the operator we are trying to solve is nondegenerate, and the wavefunction is sufficiently expressive, then

9 $$\left|\psi_{0}\right\rangle =U\left(\theta^{★}\right)\left|0\right\rangle \;w\mathrm{ith}\;\theta^{★}={\mathrm{argmin}}_{\theta \in R^{j}}\frac{\left\langle \psi \left(\theta \right)\right|H\left|\psi \left(\theta \right)\right\rangle }{\left\langle \psi \left(\theta \right)\right|\psi \left(\theta \right)\rangle }$$

where |ψ_0_⟩ is the true ground state of *H*. VQE is a widely studied hybrid quantum-classical approach for quantum chemistry on noisy intermediate-scale quantum (NISQ) devices, primarily due to its relatively shallow circuit requirements and its compatibility with present-day quantum hardware. Its simplicity instead hides scalability challenges because of the growth of expressivity of the trial wavefunction with its depth, the flatness of the corresponding energy landscape, the intensive exchange of data between quantum and classical devices, and noisy quantum devices. Despite these constraints, VQE remains a practical framework for exploring electronic structure problems on near-term quantum devices, and it provides a suitable testbed for investigating ansatz design, error mitigation strategies, and hybrid optimization techniques within the scope of this work.

### 2.3 Post-Hartree–Fock Theory

Post-HF methods systematically improve upon the mean-field Hartree–Fock approximation by incorporating electron correlation effects.^9^ Methods such as Configuration Interaction (CI),^34^ Coupled Cluster (CC),^12^ and Multi-Configurational Self-Consistent Field (MCSCF)^35^ provide more accurate and flexible descriptions of molecular electronic structure at the cost of increased computational complexity. Although CC methods (e.g., CCSD­(T)) are often considered the *gold standard* for many systems, they do not surpass Full Configuration Interaction (FCI),^34^ which remains the exact solution within a given basis set but is computationally intractable for anything beyond very small molecules.^36^ Instead, CC methods achieve high accuracy with more favorable scaling compared to the exponential cost of FCI.

For geometry optimization applications, achieving post-HF accuracy is essential to obtain reliable potential energy surfaces and accurate force evaluations. However, the steep computational scaling of traditional post-HF methods severely limits their applicability when repeated energy evaluations are required along an optimization pathway.^37^ In this context, quantum computing approaches offer a promising alternative by enabling a more compact representation of correlated wavefunctions through quantum superposition and entanglement, potentially reducing the overall computational cost of high-accuracy energy calculations within variational frameworks such as VQE.^38^

The complete active space configuration interaction (CASCI^39,40^ ) and complete active space self-consistent field (CASSCF^41,42^ ) methods represent particularly relevant targets for quantum computing implementations, as they map naturally onto variational quantum algorithms while maintaining a well-defined level of accuracy for comparison purposes.

### 2.4 Force Computation

Within the Born–Oppenheimer approximation, the molecular forces are defined as the total derivative of the electronic energy with respect to the nuclear coordinates

10 $$F=-\frac{\mathrm{dE}}{dx}$$

Let **θ** denote the variational parameters of the wavefunction (CI coefficients or, in a hybrid quantum algorithm, the circuit parameters), and let the orbitals be parametrized by a separate set of rotations **κ**. By applying the chain rule, one obtains

11 $$\frac{\mathrm{dE}}{\mathrm{dx}}={\frac{\partial E}{\partial x}|}_{\theta ,\kappa }+\frac{\partial E}{\partial \theta }\frac{d\theta }{\mathrm{dx}}+\frac{\partial E}{\partial \kappa }\frac{d\kappa }{\mathrm{dx}}$$

For a variational wavefunction, the stationarity condition

12 $$\frac{\partial E}{\partial \theta }=0$$

eliminates the CI (or circuit) response. This is true for both *variational* (CASCI, truncated CI, VQE with fixed ansatz) and *fully variational* (HF method, CASSCF) electronic structures. The key distinction lies in the second response term. Fully variational methods also satisfy

13 $$\frac{\partial E}{\partial \kappa }=0$$

because orbital rotations are optimized at every geometry. In this ideal case, eq 11 reduces to the Hellmann–Feynman^25,43^ form (with basis-set Pulay contributions^10,44^ only if the atomic orbitals themselves depend on **x**)­

14 $$\frac{\mathrm{dE}}{dx}=\underset{\mathrm{Hellmann}‐\mathrm{Feynman}}{\underset{︸}{\left\langle \Psi \left|\frac{\partial Ĥ}{\partial x}\right|\Psi \right\rangle }}+\underset{\mathrm{PulayForces}}{\underset{︸}{\left\langle \frac{\Psi }{\partial x}\left|Ĥ\right|\Psi \right\rangle +\left\langle \Psi \left|Ĥ\right|\frac{\Psi }{\partial x}\right\rangle }}$$

They need to be accounted every time the basis set depends explicitly on the atom coordinates. For variational but not fully variational methods, such as CASCI or VQE with nonrelaxed orbitals, the energy is stationary only with respect to **θ**. The orbital response term survives and gives rise to *Pulay forces*

15 $$F_{\mathrm{Pulay}}\propto \frac{\partial E}{\partial \kappa }\frac{d\kappa }{\mathrm{dx}}$$

which account for the fact that the underlying one-electron basis (and therefore the Fock and Hamiltonian matrices) changes when the nuclei move. Physically, Pulay terms correct the Hellmann–Feynman force to ensure that the electronic state remains variationally optimal with respect to the orbital manifold. This is why CASCI, truncated CI, and fixed-ansatz VQE do *not* admit a pure Hellmann–Feynman gradient: even though the wavefunction is variational in the CI (or circuit parameters) space, it is not variational in the orbital rotation space.^45^

## 3 Hybrid Quantum-Classical Implementation in the Literature

Hybrid quantum-classical approaches to molecular geometry optimization replace the electronic-structure step of a classical optimizer with a variational quantum solver, while keeping the nuclear update on the classical side. Early demonstrations adopted the simplest workable loop by obtaining a variational ground state at a trial geometry using VQE, approximate nuclear forces via finite differences of nearby single points, and update coordinates with a standard optimizer. Although straightforward, this architecture immediately raised two methodological issues central to robust geometry optimization on near-term hardware: *force quality*, i.e., whether gradients are formally consistent with the Hellmann–Feynman/Pulay^10,25,43,44^ framework in a variational setting, and *landscape navigation*, i.e., how to maintain stable convergence on a nonconvex variational energy landscape across a sequence of geometries.

### 3.1 Force Evaluation

On the theoretical side, hybrid derivative formulations clarified how Hellmann–Feynman contributions and wavefunction-response (Pulay-like) terms should be handled within variational circuits and multistate settings, providing a route to analytic forces beyond naive finite differences.^46,47^ While several studies still rely on finite differences for practicality on NISQ devices, these analyses established the conditions under which gradients are formally correct (state consistency at displaced geometries) and how response terms can be incorporated in principle, thereby informing the design of more robust geometry update loops.

### 3.2 PES Exploration

Delgado et al. formulated a variational algorithm in which circuit and Hamiltonian parameters are co-optimized to locate equilibrium structures directly on the Born–Oppenheimer surface, demonstrating end-to-end geometry optimization beyond isolated energy evaluations.^20^ Complementary efforts explored quantum-driven molecular dynamics and PES traversal using variational states with explicit gradient handling, concluding that consistent forces are essential for stable structural updates.^46,47^ For electronically challenging regions (near-degeneracies, conical intersections), hybrid quantum-classical algorithms have been proposed to navigate multisurface features and track minima in the presence of strong state interactions, illustrating how hybrid strategies can be extended to more intricate PES topologies relevant to relaxation paths.^48^

### 3.3 Beyond Vanilla VQE-Based

Beyond fixed-ansatz VQE, *ansatz-adaptive and active-space* strategies are increasingly used to stabilize PES navigation. ADAPT-style procedures^49–52^ dynamically grow the ansatz via a gradient-based operator-selection criterion, mitigating overparameterization; subsequent work has assessed and adapted this construction specifically for smooth PES traversal.^27,53,54^ An alternative route that avoids high-dimensional classical parameter optimization is the contracted quantum eigensolver (CQE),^55–61^ which solves a contracted form of the Schrödinger equation directly on the device, sidestepping both deep phase-estimation circuits and the variational landscape that plagues fixed-ansatz VQE. Relatedly, Mizukami et al.^21^ argue that fully variational hybrid quantum-classical algorithms such as Orbital-Optimized UCC^62^ favor structure optimization on quantum devices by co-optimizing orbitals and circuit parameters. Quantum embedding and quantum analogues of CASSCF further extend this active-space strategy by treating only the chemically relevant subspace on the quantum device, which is particularly attractive when structural relaxation requires multireference character.^41,63–65^ More recent embedding formulations explicitly target geometry optimization and co-optimization by coupling DMET-like fragmentation[Fn fn3] with variational solvers to reduce qubit counts and classical-quantum nesting costs, bringing routine structural refinement closer to practical scales.^22^

From these developments, a general tendency appears. For near-term devices, *finite-difference forces* remain the most common pragmatic choice in geometry loops, but must be deployed with care (ensuring state consistency at displaced geometries and controlling stochastic/shot noise) to avoid bias in updates. *Analytic-gradient frameworks* are maturing and clarify how to include response terms, positioning hybrid quantum-classical geometry optimization on firmer theoretical ground as hardware and measurement strategies improve. On the *navigation* side, the nonconvex nature of variational landscapes necessitates warm starting and, when needed, multistart or branching to escape local minima, while *ansatz adaptivity* and *embedding* provide scalable routes to maintain accuracy across a sequence of geometries.

## 4 QGO: Quasi-Adiabatic Hybrid Classical-Quantum Geometry Optimization

As briefly introduced in the previous sections, the geometry optimization procedure has the objective of identifying the equilibrium nuclear configurations by iteratively descending the PES toward stationary points of the electronic ground-state energy. Once the electronic energy is obtained through a variational quantum algorithm at a given molecular geometry, nuclear coordinates are updated based on estimates of the energy gradient until convergence criteria are satisfied, e.g., molecular gradients or bond/angle not refined. In this framework, forces are evaluated from single-point electronic structure calculations at displaced geometries, and an effective combination of energy evaluation, gradient estimation, and wavefunction preparation is required to achieve both accuracy and stability along the optimization trajectory.

In this section, we present our proof-of-concept hybrid quantum-classical method for structure optimization, namely *QGO*. The method presented here integrates three key components. First, nuclear forces are computed through finite-difference approximations, leveraging variationally optimized electronic energies at slightly perturbed geometries. Second, an *adiabatic* wavefunction-tracking strategy is employed to accelerate convergence by reusing the optimized state from the previous step whenever the PES evolves smoothly. Third, a complementary *nonadiabatic* exploration strategy is introduced to prevent variational optimization from becoming trapped in local minima. The combination of these elements provides a proof-of-concept algorithm that is flexible, reliable, scalable, and capable of achieving reasonably accurate equilibrium geometry for different molecular systems. The overall procedure is shown in a schematic and simplified diagram in Figure 1. The following subsections detail these three components of the geometry optimization protocol.

**Figure 1:**
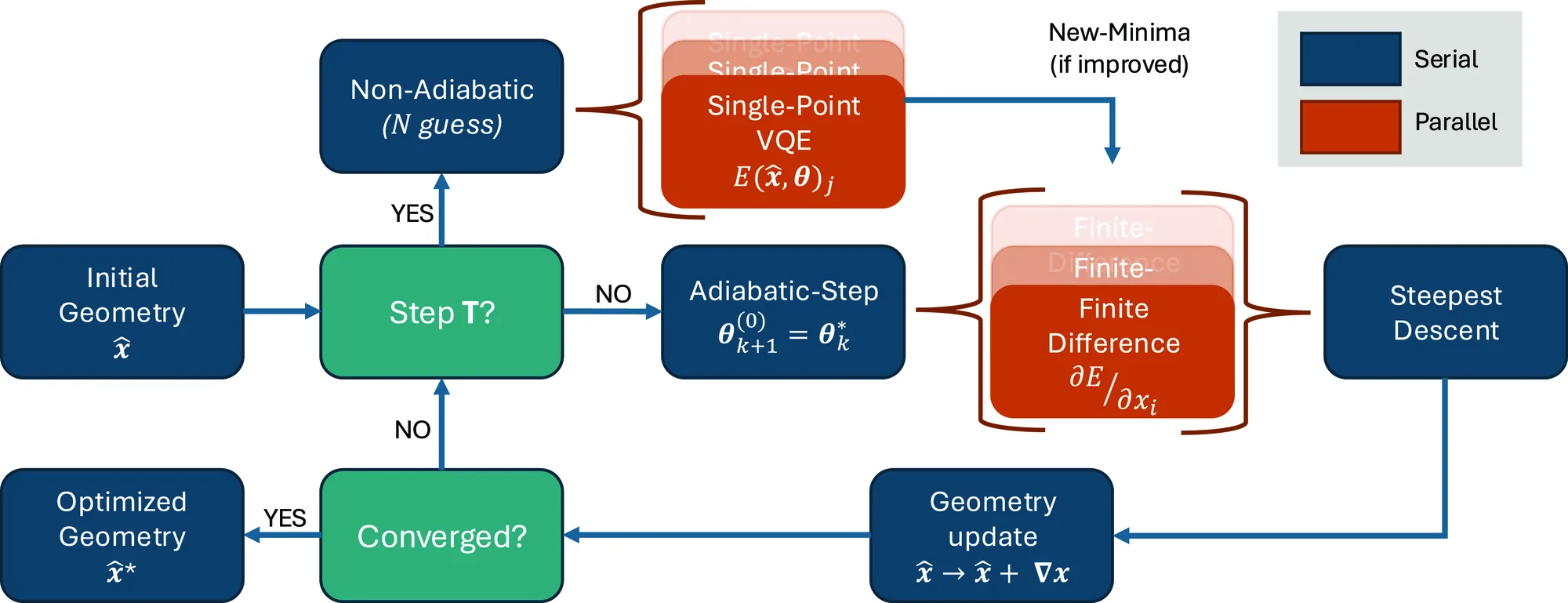
Quasi-Adiabatic Quantum Geometry–Optimization pipeline scheme.

To clarify the distinction between classical and quantum computation, we specify the division of computational work between classical and quantum resources in the QGO framework. All SCF calculations, molecular integral generation, Fermion-to-qubit mapping, nuclear coordinate updates, geometry convergence checks, and finite-difference gradient assembly are performed on classical hardware. The quantum device, represented here by a GPU-accelerated statevector emulator, is employed exclusively for evaluating electronic energies via the VQE algorithm at specified nuclear configurations: once at the central geometry per optimization step and once per displaced geometry for force evaluation.

### 4.1 Finite-Differences approach

In the context of molecular geometry optimization, the evaluation of nuclear forces traditionally relies on the computation of energy gradients with respect to nuclear coordinates, ∂*E*/∂*x*_*i*_, which can be estimated using finite-difference formulas. In particular, given an initial geometry **x**, for a given coordinate x_*i*_, the corresponding derivative in the finite difference framework is defined as

16 $$\frac{\mathrm{dE}}{dx_{i}}=\frac{E\left(x_{i}+\delta \right)-E\left(x_{i}-\delta \right)}{\left|2\delta \right|}$$

where δ is the nuclear displacement applied on the *i*-th nuclear coordinate, x_*i*_. This strategy implicitly incorporates both Hellmann–Feynman^25,43^ and Pulay-like^10,44^ effects, because the total energy difference reflects all contributions coming from the change in the electronic problem, regardless of their origin. The finite-difference strategy provides a simple and implementation-friendly estimate of the forces, at the price of requiring multiple energy evaluations and ensuring that each displaced geometry is sufficiently reoptimized.

Within a VQE framework, the molecular Hamiltonian *Ĥ*(**x**) is constructed from a given nuclear configuration **x**, and the electronic ground state is approximated by a parametrized wavefunction |ψ­(**θ**; **x**)⟩, whose parameters **θ** are optimized according to the variational principle (introduced in Section 4). Once the optimal parameters **θ** ^★^ are obtained, the ground-state energy is given by

17 $$E_{0}\left(x\right)=\frac{\left\langle \psi \left(\theta ;x\right)\right|Ĥ\left(x\right)\left|\psi \left(\theta ;x\right)\right\rangle }{\left\langle \psi \left(\theta ;x\right)\right\rangle }$$

A naive finite-difference implementation of the nuclear gradient proceeds by introducing a displacement ± *d*_*i*_ along the *i*-th coordinate and constructing the perturbed Hamiltonians *Ĥ*_±_ = *Ĥ*(**x** ± *d*_*i*_). The derivative is then approximated as

18 $$\frac{dẼ\left(x\right)}{dx_{i}}=\frac{{Ẽ}_{+}-{Ẽ}_{-}}{2d_{i}}=\frac{\langle \psi \left(\theta^{★};x\right)|{Ĥ}_{+}\left|\psi \left(\theta^{★};x\right)\right\rangle -\left\langle \psi \left(\theta^{★};x\right)\right|{Ĥ}_{-}\left|\psi \left(\theta^{★};x\right)\right\rangle }{2d_{i}}$$

where the same optimized wavefunction |ψ­(**θ** ^★^; **x**)⟩ is used for all three geometries (central, + and −). This is a reasonable approximation under the assumption that the wavefunction |ψ­(**θ** ^★^; **x**)⟩ is close enough to the true ground state.

However, this approach is theoretically inconsistent; in fact, by construction, |ψ­(**θ** ^★^; **x**)⟩ is the ground state of *Ĥ*(**x**), but it is generally *not* the ground state of *Ĥ*_±_. As a consequence, the resulting forces might neglect wavefunction-response contributions and are not consistent with the Hellmann–Feynman framework,^25,43^ nor with the Pulay corrections^10,44^ that arise when the basis (or ansatz) depends on nuclear coordinates. The theoretically correct finite-difference expression requires two independent ground-state optimizations

19 $$\left|\psi_{+}\right\rangle =\mathrm{GS}\left[{Ĥ}_{+}\right],\;\left|\psi_{-}\right\rangle =\mathrm{GS}\left[{Ĥ}_{-}\right]$$

which yield

20 $$\frac{\mathrm{dE}\left(x\right)}{dx_{i}}=\frac{E_{+}-E_{-}}{2d_{i}}=\frac{\left\langle \psi_{+}\right|{Ĥ}_{+}\left|\psi_{+}\right\rangle -\left\langle \psi_{-}\right|{Ĥ}_{-}\left|\psi_{-}\right\rangle }{2d_{i}}$$

representing the true finite-difference gradient. This formulation is fully consistent with the variational principle and incorporates the correct electronic response to nuclear displacements. In practice, however, performing two fully independent VQE optimizations for each displaced geometry may be computationally demanding and potentially unstable, as the two optimizations can follow different variational trajectories and converge to states of unequal quality.^14^

A practical strategy is therefore to initialize both optimizations from the central-geometry wavefunction parameters, i.e., setting **θ** = **θ** ^★^ for the + and—calculations. This *warm-start* approach significantly reduces computational cost while ensuring that the perturbed Hamiltonians are minimized with respect to their own electronic wavefunctions, thus recovering a consistent definition of the finite-difference gradient and avoiding the theoretical inconsistency of the naive scheme. The warm start strategy provides a physically sound balance between accuracy and efficiency, and it represents the appropriate implementation for finite-difference force evaluation within variational quantum simulations of molecular systems.

### 4.2 Adiabatic Search for Accelerated Geometry Optimization

In molecular geometry optimization with VQE, when structural changes between successive optimization steps are sufficiently small, it is advantageous to adopt an *adiabatic* search strategy, in which the converged wavefunction from the previous geometry is used as the initial guess for the next single—point calculation. This idea is loosely inspired by the concept of adiabatic continuity on a PES, which intuitively states that *if the molecular geometry is perturbed only slightly, the ground state at the new geometry is expected to remain close, in parameter space, to the ground state at the previous geometry*. Given the optimal parameters **θ**_*k*_^★^ at geometry **x** ^(k)^, the next VQE iteration is initialized as

21 $$\theta_{k+1}^{\left(0\right)}=\theta_{k}^{★}$$

For sufficiently small nuclear displacements ∥**x** ^(*k*+1)^ – **x** ^(*k*)^ ∥ ≪ 1, the energy can be expanded as

22 $$E\left(\theta_{k+1}^{★};x^{\left(k+1\right)}\right)\approx E\left(\theta_{k}^{★};x^{\left(k\right)}\right)+O\left(∥x^{\left(k+1\right)}-x^{\left(k\right)}∥\right)$$

which justifies a warm-start initialization, since the optimal solution at step *k* + 1 lies near **θ**_*k*_^★^ in parameter space, enabling faster convergence. Operationally, the adiabatic strategy reuses **θ**_*k*_^★^ as the initial guess for the VQE optimization at geometry **x** ^(*k*+1)^, thereby reducing the number of classical optimization iterations.

By avoiding repeated random or Hartree–Fock initializations, the adiabatic strategy keeps the classical optimizer in the correct basin of attraction on the variational landscape, typically reaching convergence in a small number of iterations and reducing the total computational cost by up to an order of magnitude. This behavior reflects the empirical smoothness of the PES and the expected continuity of the electronic ground state for small structural deformations. However, the adiabatic strategy remains intrinsically local i.e., it efficiently follows a single branch of the PES, but cannot prevent convergence to sub-optimal local minima in the presence of strong landscape nonconvexity or qualitative changes in the electronic structure. For this reason, the search remains adiabatic only when the PES evolves smoothly, and we introduce an additional nonadiabatic step to check whether we are in this condition, trying to ensure robustness beyond the local PES topology. A pictorial representation of the adiabatic procedure is depicted in panel Figure 2(a).

**Figure 2:**
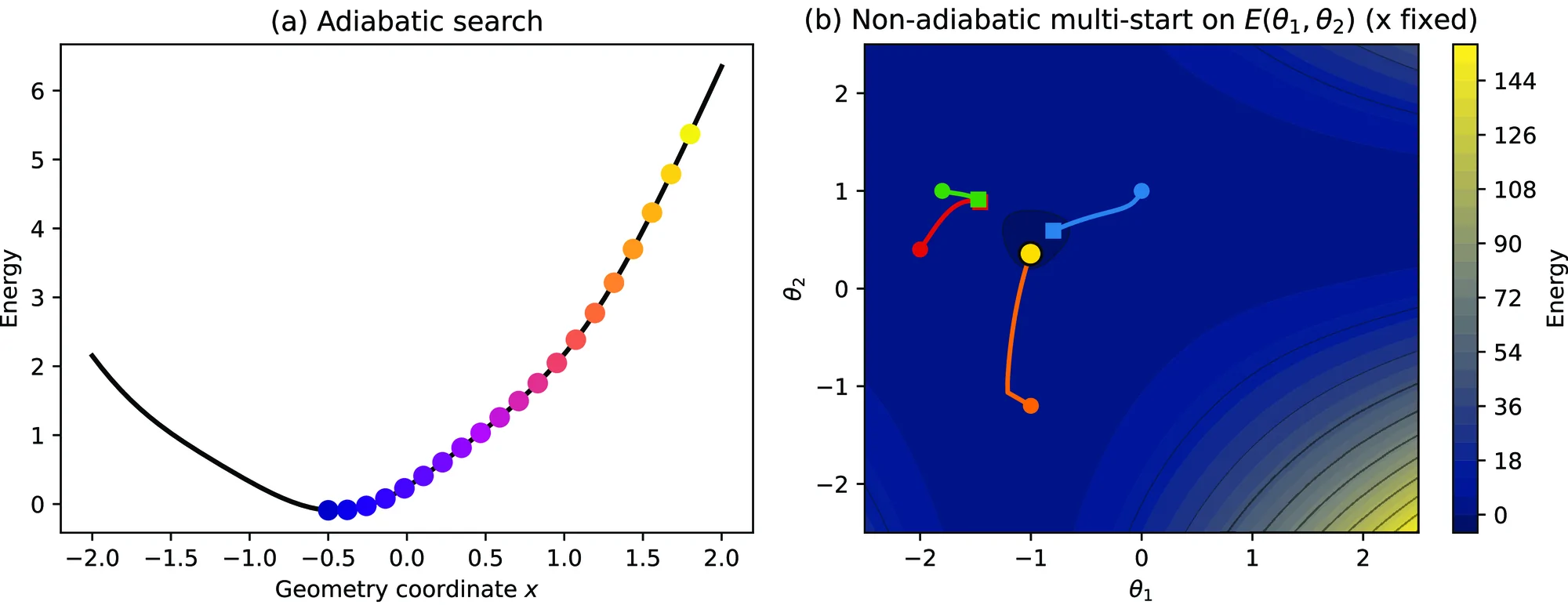
Adiabatic versus nonadiabatic search in VQE-driven geometry optimization. In panel (a), a warm-start (adiabatic) strategy tracks a single valley of the potential energy surface (PES), enabling rapid local convergence for small structural variations. In panel (b), when this strategy risks trapping in a local minimum, a nonadiabatic multistart exploration is performed at fixed nuclear geometry: multiple trajectories are initialized on the variational energy landscape *E*(θ_1_, θ_2_), and the lowest-energy solution is selected. Circles denote initial points, while squares indicate the corresponding optimized end points.

### 4.3 Nonadiabatic Search for New Local Minimum Identification

To overcome the limitations imposed by the nonconvex nature of the variational optimization landscape, in which VQE procedures can become trapped in suboptimal local minima,^14^ we incorporate a *potentially nonadiabatic* search strategy designed to improve minima exploration. At predefined intervals *T* along the geometry optimization, instead of warm-starting from the previously converged wavefunction, the algorithm generates an ensemble of initial parameter configurations

23 $$\left\{\theta^{\left(1\right)},...,\theta^{\left(N\right)}\right\}$$

obtained either by perturbing the previous solution or by fully randomized initialization in the range θ_*k*_ ∈ (−π, π]. Each candidate is variationally optimized, and the retained state is chosen according to

24 $$\theta_{k}^{★}=\underset{j\in \left\{1,...,N\right\}}{\arg\min}E\left(\theta^{\left(j\right)};x^{\left(k\right)}\right)$$

corresponding to the branching energy rule

25 $$E_{\mathrm{branch}}\left(x^{\left(k\right)}\right)=\min_{j}\left\{E\left(\theta^{\left(j\right)};x^{\left(k\right)}\right)\right\}$$

This branching mechanism enables, if exploiting enough expressive and accurate circuit, and ideal global exploration of the PES and mitigates the risk of converging to suboptimal local minima, particularly in the presence of complex landscapes or quasi-degenerate states.

Operationally, the nonadiabatic step samples distinct regions of the parameter landscape by launching *N* independent VQE optimizations

26 $$\theta^{★\left(j\right)}=\arg\min_{\theta }E\left(\theta ;x^{\left(k\right)}\right),j=1,...,N$$

and selects the lowest-energy solution as the new reference state for continuing the geometry optimization. After this branching phase, the algorithm reverts to the adiabatic mode to efficiently refine the geometry, reactivating nonadiabatic exploration every *T* steps. The term *potentially nonadiabatic* reflects the fact that the evolution remains predominantly adiabatic, but temporarily deviates from the adiabatic path whenever a more favorable minimum is discovered. This hybrid adiabatic/nonadiabatic strategy balances global robustness with local efficiency, making it well-suited for challenging molecular systems where following a single adiabatic trajectory is insufficient to guarantee reliable ground-state tracking across the VQE. A pictorial representation of the nonadiabatic procedure is depicted in panel Figure 2(b).

It is worth emphasizing that the geometry, **x** ^(*k*)^, remains fixed during the nonadiabatic branching step. While the adiabatic update explores the PES along nuclear coordinates, the nonadiabatic procedure explores instead the variational landscape *E*(**θ**; **x** ^(*k*)^) at fixed geometry, in order to escape local minima of the VQE energy surface.

### 4.4 Molecular Orbital Phase Consistency

A critical prerequisite for the warm-started reoptimization of displaced-geometry VQEs (eqs 19 and 20) is the consistency of the molecular orbital phase convention between the central and displaced SCF calculations. Molecular orbitals are defined only up to an overall phase: ϕ_*i*_ and −ϕ_*i*_ and yield identical energies and densities in classical quantum chemistry, but produce different qubit Hamiltonians after Jordan–Wigner mapping (eqs 2 and 3), because the Pauli string coefficients are derived directly from the signed one- and two-electron integrals. A phase flip in a displaced-geometry orbital therefore generates a qubit Hamiltonian that is numerically inconsistent with the one at the central geometry, causing the warm-started VQE to converge to a physically inconsistent state and producing spurious contributions to the finite-difference gradient. This issue has a bigger impact for delocalized π-orbitals, where the SCF solver may select opposite phase conventions at geometries differing by an infinitesimal displacement δ. To prevent this, QGO applies an orbital-coherence correction after each displaced-geometry SCF: the leading coefficient of each displaced MO is compared with the corresponding central-geometry MO, and the displaced orbital is flipped if the signs disagree. The corrected orbitals are then used to assemble the displaced qubit Hamiltonian. This step introduces no additional quantum circuit evaluations and operates solely on the classical SCF output already available within the finite-difference workflow.

## 5 Framework Structure

In this section, we report the implementation and coding part of the algorithm, dividing them between how we deal with the chemistry side, i.e., the generation of the molecules and associated operators, and the computational side, i.e., how we simulate the circuit, how the evaluation is computed, and also, the technical side in terms of parallelization routines.

### 5.1 Quantum Chemistry Backend

Aside from the computational setting, in terms of convergence thresholds, number of macro- and micro-iteration, frequency of *potentially nonadiabatic* searches or update step, for citing some of them, the only inputs that the *QA–q–GeomOpt* algorithm should (at least) ideally receive are the initial coordinates **x**, the basis set, and the type of circuit used. From the point of view of the circuit, the next section will address it. In terms of chemistry, we wanted a code, and therefore an algorithm, that was agnostic to the quantum chemistry backend used.

For the proof-of-concept, we defined the whole pipeline using *PySCF* ^66–68^ as quantum chemistry backend, which was both constrained due to the *QuAQ* ^69^ code base dependency on it but also for its accessibility. In term of implementation point of view, the chemistry backend is used for the extraction of the one- and two-body integrals. In particular, for PySCF, using the CASCI constructor mcscf.CASCI and the two methods get_h1eff and get_h2eff, we can extract the effective one- and two-integrals, as well as the core energy. Then, using Qiskit-Nature ElectronicEnergyProblem, we use the method from_raw_integrals to obtain the FermionicOperator associated with the electronic Hamiltonian to be solved. Additionally, PySCF allows to build molecules both with *Cartesian coordinates*, defined by a list of tuple atom, *x*,*y*,*z* , and *Internal coordinates* or *z*-matrix, defined in our case by a template string in which the coordinates are replaced by placeholders and a dictionary containing the value associated with each placeholder coordinate.

### 5.2 Quantum Computing Simulation Backend

For the circuit simulation, we implemented two different quantum computing backend: Qiskit statevector,^70^ and CudaQ,^71^ statevector and GPU simulator. The whole backbone is defined and integrated using some revised QuAQ functionalities, adapted for the geometry optimization task, like the VQE algorithm, the symmetry penalty operators (*N̂*_*e*_, *Ŝ*_*z*_, and *Ŝ* ^2^), and the effective operator construction. The underlying management of the Fermionic operators and qubit operators is defined using Qiskit-Nature^72^ FermionicOperator and Qiskit SparsePauliOp, respectively. If the backend chosen for the simulation is Qiskit, then no additional transformation is required. If the backend is CudaQ, then all the qubit operators are converted, using QuAQ.tools.converters, from SparsePauliOp to CudaQ SpinOperators. The main difference between the two implementations is the circuit object. In fact, Qiskit use a QuantumCircuit dataclass, which, once instantiated allows for further circuit manipulation. CudaQ, instead, uses a cudaq.kernel decorator applied to a Python method that builds the quantum state given a desired set of specifics and the circuit parameters. The second structure is more constrained, requiring also more care in the setup of the method, especially for parallelization. Additionally, in order to perform an expectation value, contrary to Qiskit in which a statevector can be converted into a complex array and contracted to the matrix representation of the qubit operator, CudaQ has a specific method, namely cudaq.observe, which requires an operator defined as SpinOperator, a circuit kernel, and the set of parameters and arguments required to build the associated circuit.

### 5.3 Intranode Parallelization

Force calculations via finite differences require energy evaluations at displaced geometries. Given a geometry with 3*N*_atoms_ Cartesian degrees of freedom, the requirement of a single-step structure optimization is 6*N*_atoms_ additional VQE calculations, if finite differences method is used. Switching to internal coordinates, the evaluation requirement drops 6*N_atoms_* – 12, given 3*N*_atoms_ – 6 original degrees of freedom in the structure of a general nonlinear molecule. We implement parallel evaluation strategies to distribute these calculations across available computational resources. The parallel framework divides force component calculations among independent workers, each performing complete VQE optimizations for their assigned displaced geometries. In particular, a single task is defined with initial geometry, cords, coordinate to which apply the displacement, i, value and sign of the displacement, d and sign, respectively, the method for building the displaced geometry, builder, the evaluation method, evaluation, the initial variational parameters, psi_0, and the optimization strategy, optimization_procedure. We report here the structure of a gradient worker Scheme 1.

**Scheme 1:**
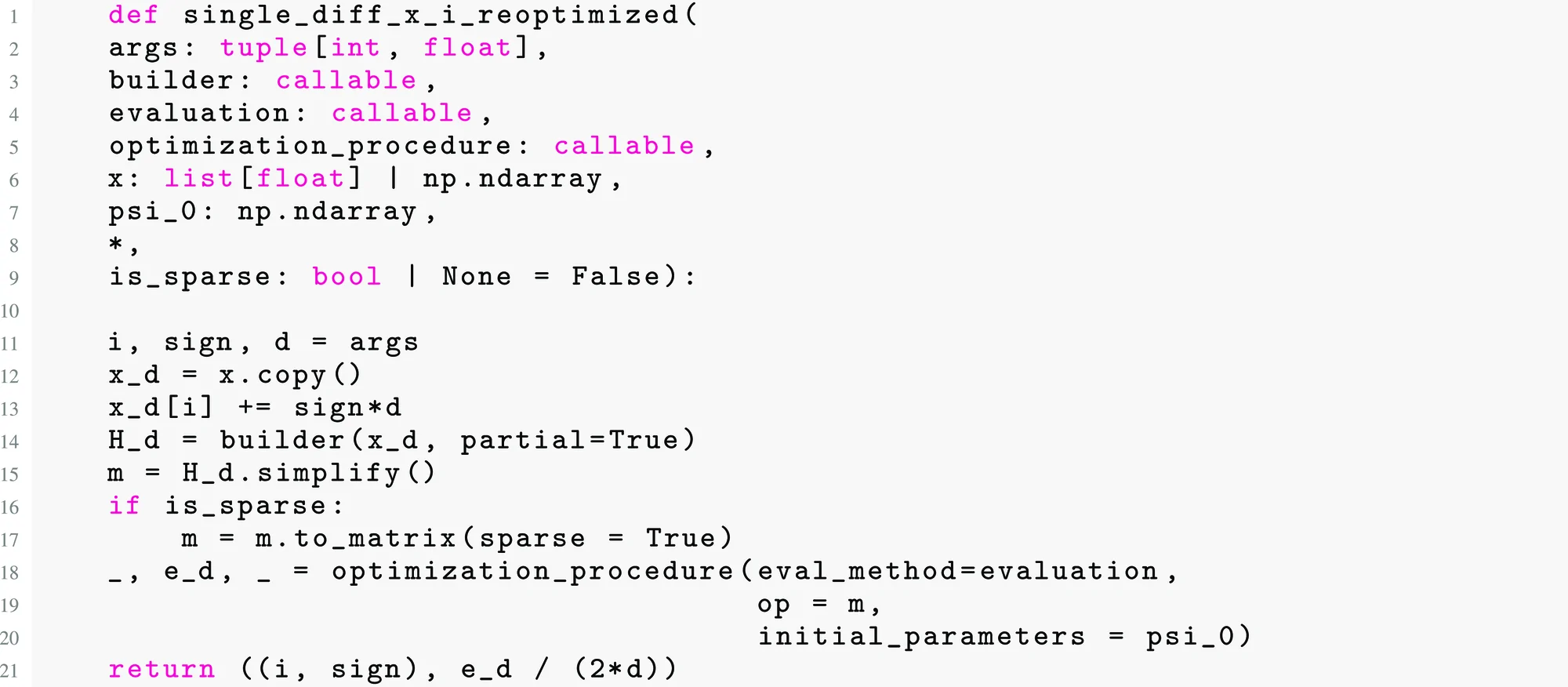
Snippet 1: Single Perturbed Geometry Energy Evaluator

Here, every argument except the task-specific info, position, and displacement is fixed using the Python partial wrapping method.

At this point, for all the coordinates, for both Cartesian and internal, and for all the displaced directions, for both positive and negative displacement, the associated task is defined as a tuple (i, sign, d) and evaluated in parallel using joblib as a multiprocessing manager. Then, all of the evaluations are collected, and the gradient vector is defined.

Additional parallelization is implemented during the nonadiabatic new minima search, which is performed at the beginning of the PES minimization process and then after every *T* step. In this case, the worker has a more compact and simpler structure compared to the one for the single perturbed energy evaluation. In particular, each worker receives a fresh new set of random parameters, initial_parameters, an operator to minimize, op, and an evaluation method, eval_method. Internally then, it calls a scipy.optimize.minimize instance as follows Scheme 2:

**Scheme 2:**
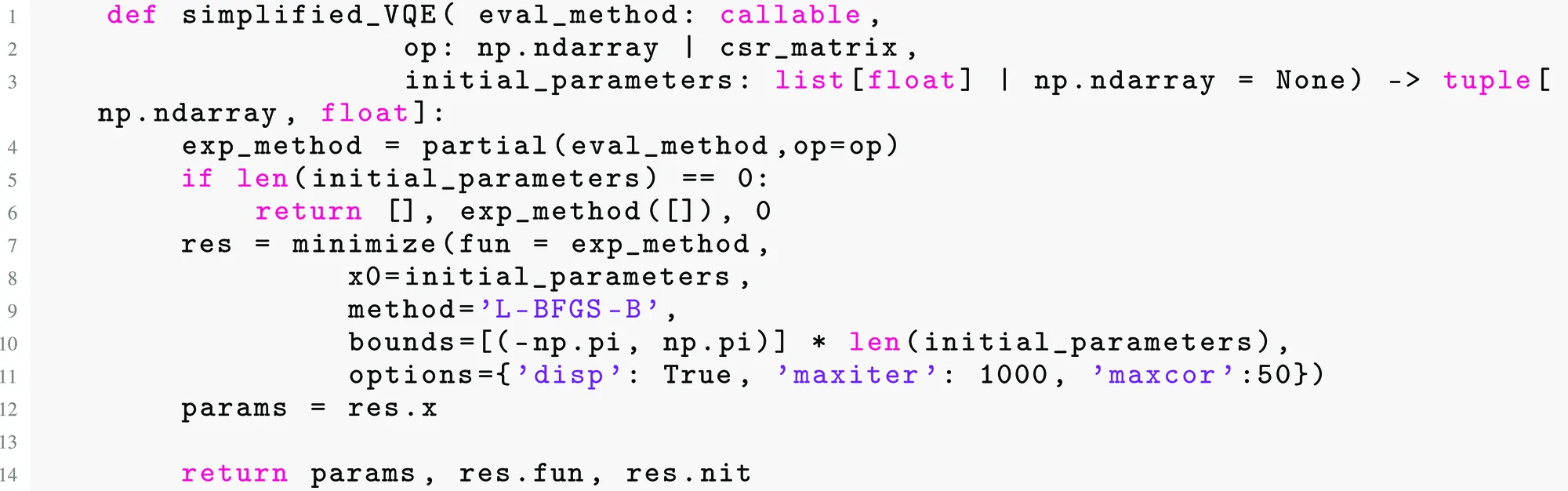
Snippet 2: Parallel Non-Adiabatic VQE Worker

Once again, given the number of workers, each worker receives a task that is only composed of the set of initial parameters, while the other arguments are fixed using Python partial.

### 5.4 Geometry Update Strategy

Molecular geometries are updated using gradient descent methods with adaptive step sizes.^47^ While more sophisticated optimization algorithms (such as quasi-Newton methods or trust-region approaches) are available in the literature, we employ simpler schemes to maintain transparency and facilitate analysis of convergence behavior.

## 6 Computational Setup

After illustrating the theoretical and methodological background of our quantum geometry optimization procedure, in the following section, we introduce the computational setup and the molecular system that have been approached with our quantum structure optimization routine. We include analyses of molecules obtained from DKs^73–76^ —descriptor sets that can be interpreted as substructure search patterns or structural fragments relevant for drug-discovery applications.

### 6.1 DompeKeys Selected

Functional groups, such as imines, amides, and aldehydes, play a central role in both structural chemistry and medicinal design. Their presence strongly influences molecular conformation, hydrogen-bonding capacity, and electronic distribution, which in turn modulate pharmacophoric behavior and chemical reactivity. Within the fragment dataset, these structures occur frequently and represent chemically diverse yet computationally accessible systems.

#### 6.1.1 Imines

Imines are characterized by a C = N bond, formally derived from the condensation of carbonyl compounds with primary amines. They serve as key intermediates in many biochemical transformations, including enzymatic transamination[Fn fn4] and Schiff-base formation[Fn fn5], and appear in drug scaffolds where reversible C=N bonding can modulate conformational flexibility or metal-binding properties.^77–79^ Their moderate size makes them ideal prototypes for evaluating how quantum methods handle delocalized π-systems during geometry optimization. As the first test molecule, we selected the *Methylene Imine* ^80^ defined as H_2_C = NH^81^ with initial geometry optimized at the mean-field level with Restricted-HF and STO-3g basis set, fetched from the cccbdb database. The initial geometry is shown in Figure 3 and the bonds/angle in Table 2.

**Figure 3:**
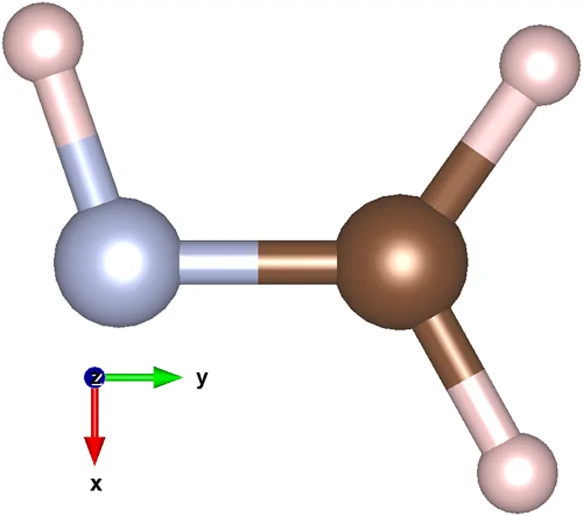
Methylene Imine initial geometry optimized with Restricted-HF and STO-3g basis set.

#### 6.1.2 Aldehydes

Aldehydes feature a polar carbonyl group with an electrophilic carbon center and a strongly polarized C–H bond, which makes them central building blocks in oxidation, condensation, and polymerization reactions, relevant also in the context of drug metabolism and covalent inhibitor design.^82^ In this set, aldehydes provide a baseline to assess whether our geometry optimization procedure can reproduce consistent trends for polar molecules. As test molecules, for the aldehydes, we selected *acetaldehyde*, defined as CH_3_CH=O, with initial geometry optimized at the mean-field level with restricted HF and STO-3g basis set, fetched from the http://cccbdb.nist.gov/cccbdb database. The initial geometry of acetaldehyde is shown in Figure 4, and the bonds/angle are shown in Table 3.

**Figure 4:**
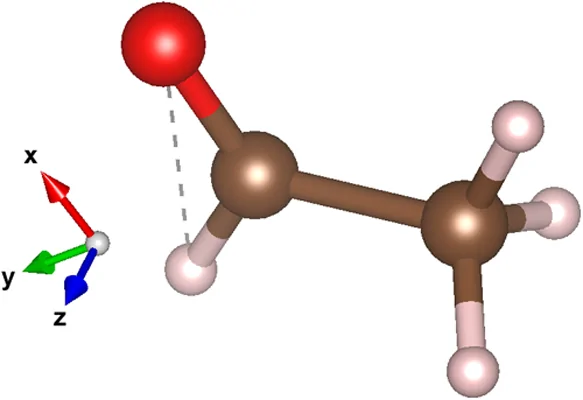
Acetaldehyde initial geometry optimized with Restricted-HF and STO-3g basis set.

#### 6.1.3 Amides

Amides are among the most ubiquitous functional groups in organic and biological chemistry, essential in the construction of peptides and proteins. Their stability arises from strong resonance between the carbonyl and the adjacent nitrogen lone pair, leading to partial double-bond character and restricted rotation about the C–N bond.^83–86^ These features test quantum algorithms against shallow potential energy surfaces and small energy gradients, typical in biological systems. For this functional group, we have selected acetamide, which is an organic compound with the formula CH_3_CONH_2_, derived from ammonia and acetic acid, commonly known for its use as an industrial solvent.^87^ The initial geometry is optimized at the mean-field level with Restricted-HF and STO-3g basis set, fetched from the http://cccbdb.nist.gov/cccbdb database. The initial geometry of acetamide is shown in Figure 5 and the bonds/angles in Table 4.

**Figure 5:**
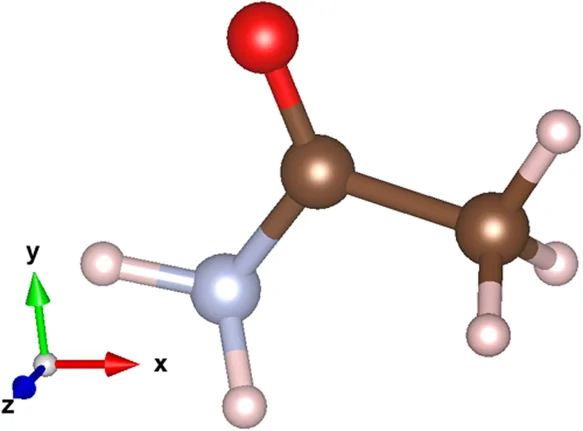
Acetamide initial geometry optimized with Restricted-HF and STO-3g basis set.

Together, these three families of compounds span a representative range of bonding situations, from delocalized π-conjugation in imines and amides to polar σ–π separation in aldehydes, providing an ideal chemical landscape for benchmarking the behavior of structure optimization algorithms across distinct electronic regimes.

### 6.2 Circuital Requirement

Given the fact that the structure refinement depends strongly on the possibility to describe as precisely as possible the largest portion of the molecular system, we define for all the DKs a moderate-sized active space consisting of 6 electrons in 6 orbitals, i.e., CAS­(6,6), and the active orbitals have been selected around the Fermi level. For all the DKs, the basis set chosen is cc-pVDZ, and the integrals on which the electronic Hamiltonians are built, according to eq 1, are defined from restricted-HF orbitals.

In terms of the circuit model selected, we selected a hardware-efficient ansatz for all the molecules. In particular, CNOT-based ladder-fashion ansatz with different depths according to the hardware-efficient ansatz studied. The circuit is defined by a layer of parametrized rotations *R*_*y*_(θ), *N*–1 CNOTs in a top-down ordered configuration, followed by another set of parametrized rotations. The number of repetitions of the rotation and entangling gates is denoted as depth, *d*. For the molecular system studied, we used a depth *d* ladder ansatz as reported in Table 1. The initial set of parameters has been selected starting from the usual randomized initialization in the range (−π, π) and HF initialization with *X* gates in correspondence with the occupied orbitals in the HF determinant.

**Table 1:** Circuital Resources Required by Our Structure Optimization Procedure for the Resolution of the Three DKs^a^

	Depth	*CNOTs*	Params
Methylene Imine	4	44	60
Acetaldehyde	4	44	60
Acetamide	4	44	60

a As reported in the paragraph, the active space is a CAS­(6,6) selected around Fermi-level corresponding to 12 qubits.

All the simulations have been run using 50 VQE as initial nonadiabatic search, 50 VQE trials, with a frequency step of 3 macro-iterations. As optimizer, we employed L-BFGS-B with maxcor = 50 and bounded parametrization in the range defined above, i.e., (−π, π).

For all the DKs, the initial geometry has been fetched from http://cccbdb.nist.gov/cccbdb database, and in particular, we started from a geometry optimized in the minimal basis set, STO-3g, at the mean-field level with Restricted-HF. In order to assess the accuracy of our method, we compared the bond length and angles between the initial geometry and the one obtained with our QGO procedure, and the exact solution obtained by optimizing the structure using CASCI with the same setup. Additionally, to validate the final structure, we also compared to the optimal distances and angles obtained by CASSCF, CCSD, and DFT with B3LYP functional. These additional calculations provide a consistent comparison across different levels of electronic-structure theory: CASSCF representing a fully variational multiconfigurational method with orbital relaxation, CCSD a nonvariational correlated wavefunction approach that closely approximates the CCSD­(T) gold standard, and B3LYP a widely adopted hybrid density-functional method whose accuracy for molecular geometries is well established, particularly when used with correlation-consistent basis sets such as cc-pVDZ.

In order to assess the agreement between the geometries obtained from the VQE optimization and the CASCI reference, the numerical precision of bond lengths and bond angles is evaluated within commonly accepted chemical accuracy limits. For reliable electronic-structure methods with medium to large basis sets, deviations smaller than approximately 0.02 Å in bond lengths and 1° in bond angles are considered chemically insignificant, while differences below 0.05 Å and 3° still indicate very good structural agreement. Therefore, all the comparisons discussed in this work are interpreted within these established precision criteria. Finally, we compare the root-mean-square deviations (RMSD) of atomic positions relative to reference structures for all of the three DK QGO-optimized structures.

## 7 Results

In this section, we report all the results obtained by our QGO procedure against the three DompéKeys selected. The results, from the point of view of accuracy, are discussed and compared against standard quantum chemistry methods involved in structure optimization. Two subsection are also dedicated to the computational efficiency of the algorithm and the challenges that the procedure still faces.

### 7.1 Methylene Imine

Table 2 reports the optimized structural parameters of methylene imine as obtained from different electronic-structure methods. The initial geometry was generated at the restricted HF level with the minimal STO-3G basis, while subsequent optimizations were carried out using the cc-pVDZ basis set to ensure a consistent level of correlation treatment. In particular, the CASCI, QGO, and CASSCF calculations employed a CAS­(6,6) active space constructed from orbitals selected around the Fermi level, as reported in the previous paragraph. The QGO procedure reproduces the CASCI equilibrium structure with excellent accuracy; bond-length deviations remain below 0.02 Å, and bond-angle differences are within 1°, which are both well inside the thresholds commonly regarded as chemically indistinguishable. The deviations between the QGO and CASCI geometries are minimal, with bond-length differences ranging from 0.003 to 0.017 Å and bond-angle variations below 1°, confirming the agreement between the two metods. The small residual variations observed between QGO and CASCI geometries can be attributed to finite-difference numerical noise and the limited stochastic precision intrinsic to variational quantum algorithms. CASSCF results, which include additional orbital relaxation, show only slightly larger discrepancies, while the CCSD and B3LYP optimizations yield marginally elongated C–H and N–H bonds, a well-known tendency of these correlated and DFT approaches, respectively.

**Table 2:** Bond Lengths and Distances between Methylene Imine^80^ Starting Geometry Obtained with **HF**, Our Geomopt Procedure (**QGO**), the Reference **CASCI** Results, with Also **CASSCF**, **CCSD**, and DFT with the **B3LYP** Functional^a^

Measure	HF	CASCI	QGO	CASSCF	CCSD	B3LYP	CRYSTAL
C1–H1	1.091	1.091	1.090	1.090	1.106	1.107	1.103
C1–H2	1.089	1.089	1.087	1.087	1.102	1.102	1.081
H3–N1	1.048	1.013	1.013	1.037	1.032	1.031	1.023
N1–C1	1.273	1.274	1.274	1.293	1.282	1.271	1.273
H1–C1–H2	115.5	116.8	116.1	116.9	116.1	115.7	116.9
H1–C1–N1	125.4	124.2	124.4	124.7	125.2	125.7	123.4
H2–C1–N1	119.1	119.0	119.5	118.4	118.7	118.6	119.7
H3–N1–C1	109.1	110.1	110.3	108.2	108.8	110.0	110.5

a Bond lengths are given in Ångstrom while angles in degrees.

Relative to the HF geometry obtained with the minimal STO-3g basis, the correlatedQGO and CASCI structures exhibit slightly longer bond lengths (by up to 0.03 Å) and small angular adjustments within 2–3°, reflecting the inclusion of electron-correlation and basis-set effects. Although these differences are modest in magnitude, they represent a quantitative improvement in the structural description.

### 7.2 Acetaldehyde

Table 3 summarizes the optimized bond lengths and bond angles for acetaldehyde obtained from different electronic-structure methods. As explained in the previous paragraph, the reference structure was computed at the CASCI level using a CAS­(6,6) active space constructed from orbitals around the Fermi level and the cc-pVDZ basis set. The QGO optimization reproduces the CASCI equilibrium geometry with high quantitative accuracy, i.e., bond-length differences lie within 0.002–0.009 Å, and angular deviations remain below 1°, indicating an almost perfect correspondence between the two approaches. Compared to the initial restricted-HF/STO-3G geometry, both CASCI and QGO yield slightly shorter C–O and longer C–C and C–H bonds, consistent with the inclusion of electron-correlation effects and the improved flexibility of the cc-pVDZ basis. CASSCF geometries, which include additional orbital relaxation, remain close to CASCI but display marginally larger bond distances, whereas CCSD and B3LYP produce the expected systematic bond elongation typical of correlated and density-functional treatments, respectively. Overall, the QGO geometry of acetaldehyde closely mirrors the multiconfigurational CASCI reference, demonstrating the reliability of the variational quantum optimization in capturing both local and global structural features of a correlated molecular systems.

**Table 3:** Bond Lengths and Distances between Acetaldehyde^82^ Starting Geometry Obtained with **HF**, Our Geomopt Procedure (**QGO**), the Reference **CASC** Results, with Also **CASSCF**, **CCSD**, and DFT with **B3LYP** Functional^a^

Measure	HF	CASCI	QGO	CASSCF	CCSD	B3LYP	CRYSTAL
H1–C1	1.104	1.104	1.101	1.107	1.121	1.124	-
C1–O1	1.217	1.208	1.207	1.217	1.212	1.208	1.216
C2–C1	1.536	1.505	1.510	1.506	1.513	1.507	1.501
C2–H2	1.085	1.090	1.090	1.089	1.101	1.099	1.08 6
C2–H3	1.087	1.081	1.081	1.093	1.105	1.104	1.114
C2–H4	1.087	1.081	1.081	1.093	1.105	1.104	-
H1–C1–O1	121.4	119.4	119.1	120.6	120.7	120.7	-
O1–C1–C2	124.3	124.6	124.8	124.7	124.5	124.8	123.9
H1–C1–C2	114.3	116.0	116.0	114.8	114.8	114.5	117.5
C1–C2–H2	110.5	109.9	110.3	110.4	110.2	110.7	-
C1–C2–H3	109.9	108.5	109.0	109.7	109.6	109.6	-
C1–C2–H4	109.9	108.5	109.0	109.7	109.6	109.6	-
H2–C2–H4	109.2	109.7	109.4	109.9	110.1	110.2	108.3
H2–C2–H3	109.2	109.7	109.4	109.9	110.1	110.2	-
H3–C2–H4	108.1	110.6	109.7	107.3	107.3	106.5	-

a Bond lengths are given in Ångstrom while angles in degrees.

### 7.3 Acetamide

Table 4 presents the optimized structural parameters of acetamide obtained with the QGO procedure in comparison with reference CASCI results and several conventional electronic-structure methods. All correlated calculations were performed with the cc-pVDZ basis set, and the multiconfigurational methods employed a CAS­(6,6) active space constructed from orbitals near the Fermi level. The QGO optimized geometry shows very close agreement with the CASCI reference, with bond-length deviations ranging between 0.002 and 0.015 Å and angular differences within approximately 1–2°, indicating that the variational quantum optimization is able to reproduce the correlated equilibrium structure with quantitative fidelity. Relative to the restricted-HF/STO-3g starting geometry, both CASCI and QGO predict a slight shortening of the C = O and N–H bonds and a moderate elongation of the C–C linkage, reflecting the inclusion of electron-correlation and polarization effects. CASSCF results, which include full orbital relaxation, remain consistent with CASCI while showing small systematic shifts in bond distances. The CCSD and DFT/B3LYP optimizations exhibit the expected trend toward marginally longer covalent bonds, a characteristic behavior of these correlated and density-functional approaches.

**Table 4:** Bond Lengths and Distances between Acetamide^83^ Starting Geometry Obtained with **HF**, Our Geomopt Procedure (**QGO**), the Reference **CASCI** Results, with **CASSCF**, **CCSD**, and DFT with **B3LYP** Functional^a^

Measure	HF	CASCI	QGO	CASSCF	CCSD	B3LYP	CRYSTAL
H1–C1	1.085	1.086	1.089	1.087	1.099	1.097	-
H2–C1	1.087	1.085	1.087	1.093	1.104	1.103	1.124
H3–C1	1.086	1.084	1.087	1.092	1.103	1.102	-
C1–C2	1.541	1.516	1.532	1.514	1.523	1.522	1.519
C2–O1	1.218	1.206	1.198	1.220	1.217	1.219	1.220
C2–N1	1.445	1.378	1.392	1.378	1.385	1.374	1.380
N1–H4	1.028	1.000	1.004	1.001	1.016	1.014	1.022
H5–N1	1.027	0.999	0.999	0.999	1.014	1.012	-
H1–C1–H2	109.0	109.3	108.7	109.2	109.3	108.8	-
H1–C1–H3	109.4	109.4	109.1	109.7	109.8	109.7	-
H2–C1–H3	108.5	108.9	108.2	107.9	108.0	107.9	-
H1–C1–C2	109.7	108.9	110.7	109.3	109.0	108.8	-
H2–C1–C2	110.4	110.0	110.2	110.3	110.3	110.1	109.8
H3–C1–C2	109.8	110.3	109.9	110.3	110.4	111.5	-
C1–C2–O1	124.5	122.5	122.7	123.3	123.4	123.2	122.9
C1–C2–N1	113.6	115.6	114.8	114.5	114.2	114.7	115.1
O1–C2–N1	121.8	121.8	122.4	122.2	122.3	122.1	122.0
C2–N1–H4	110.9	114.4	113.1	115.1	114.4	117.0	-
C2–N1–H5	112.1	118.3	114.4	118.4	117.9	121.2	-
H4–N1–H5	109.3	114.8	110.9	115.2	115.1	117.7	-

a Bond lengths are given in Ångstrom while angles in degrees.

As evident from Tables 2, 3, and 4, the geometric parameters—specifically bond lengths and bond angles—obtained through our Quantum Geometry Optimization (QGO) method demonstrate strong agreement with those computed using Hartree–Fock (HF) and post-HF methods. For completeness, we have included experimental crystal structure values from the Computational Chemistry Comparison and Benchmark DataBase (CCCBDB). However, it is crucial to emphasize that the most meaningful and rigorous benchmark for our method is achieved through comparison with Complete Active Space Configuration Interaction (CASCI) calculations Figure 6, rather than with experimental structures or full-space quantum chemical methods.

**Figure 6:**
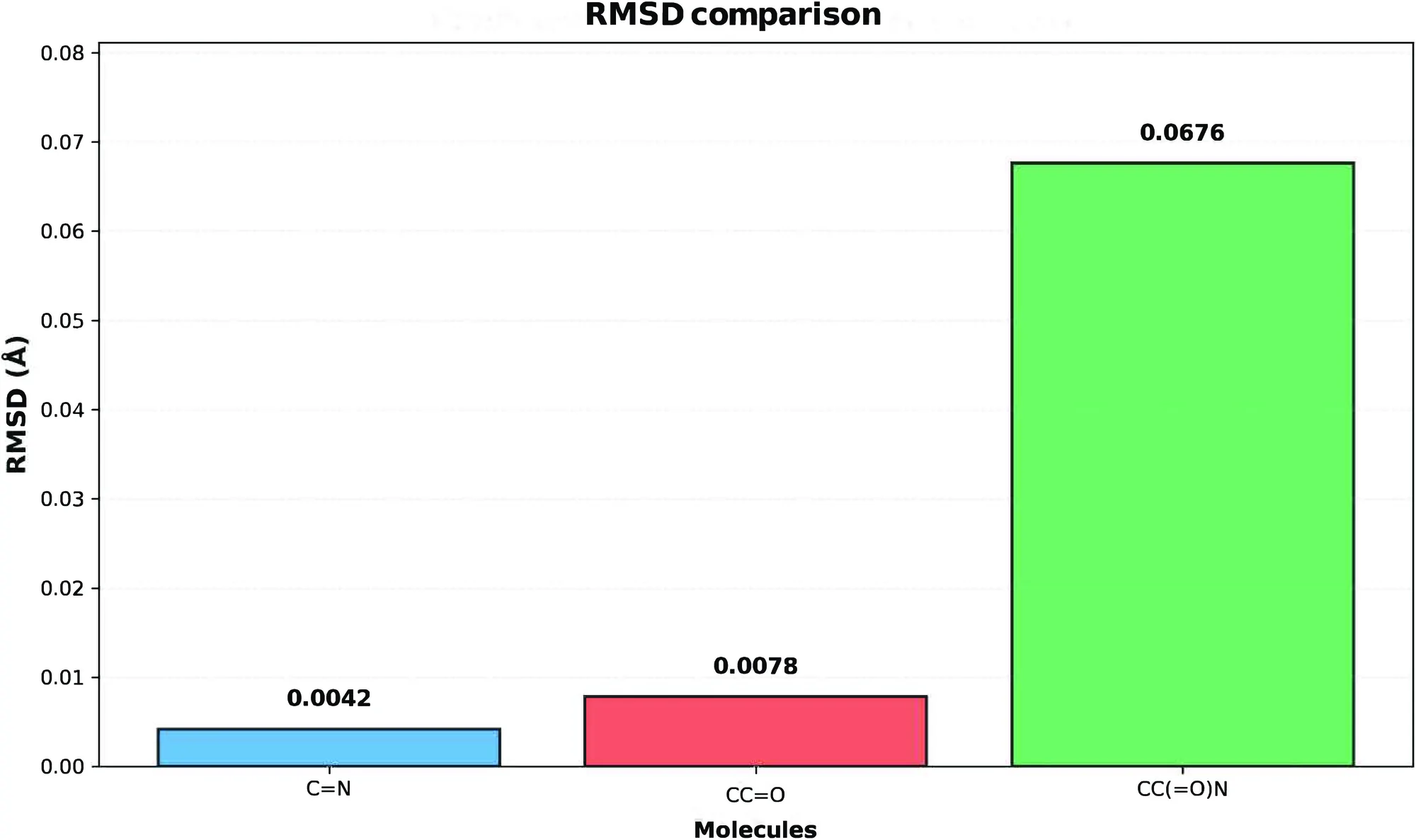
Root Mean Square Deviation (RMSD) for three different Dompé Keys fragments labeled by corresponding SMILE w.r.t. CASCI geometry prediction.

### 7.4 Computational Efficiency

We detail time analyses quantifying the computational cost of our implementation, broken down by major components: VQE energy evaluations, force calculations, and geometry updates. We discuss clock times with a parallelized approach against reference calculations and the current computational overhead Figure 7.

**Figure 7:**
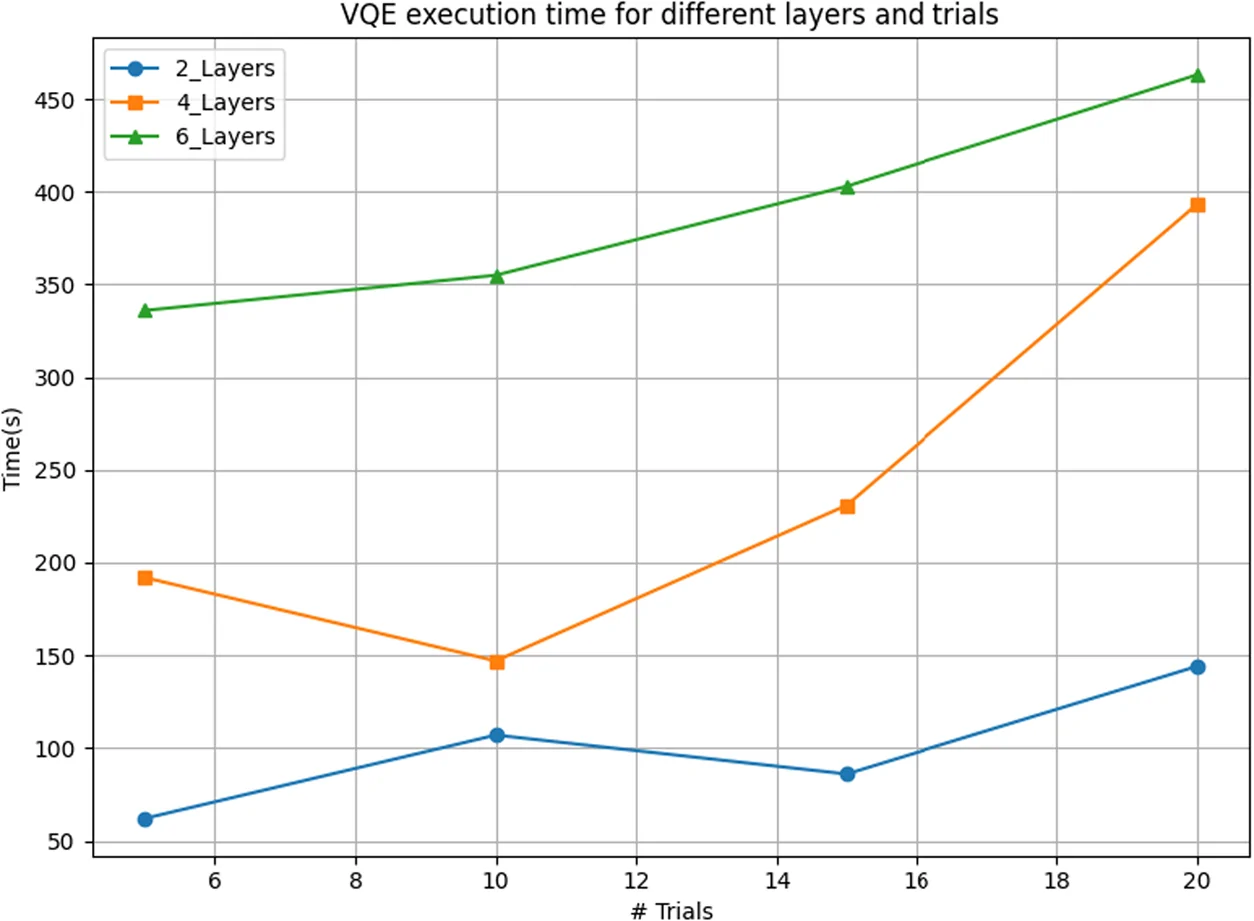
Wall-clock time reduction achieved through parallel execution of simulations for varying Hardware-Efficient Ansatz depths (number of gate layers, see legend) and multiple random initializations. This approach facilitates the selection of the lowest-energy solution across the explored Hilbert space. Sequential execution is not shown due to scale.

GPU acceleration performance is characterized across different hardware platforms, demonstrating the computational performance of the approach on the accessible computing resources. We provide scaling analyses indicating how computational requirements grow with system size, Figure 8.

**Figure 8:**
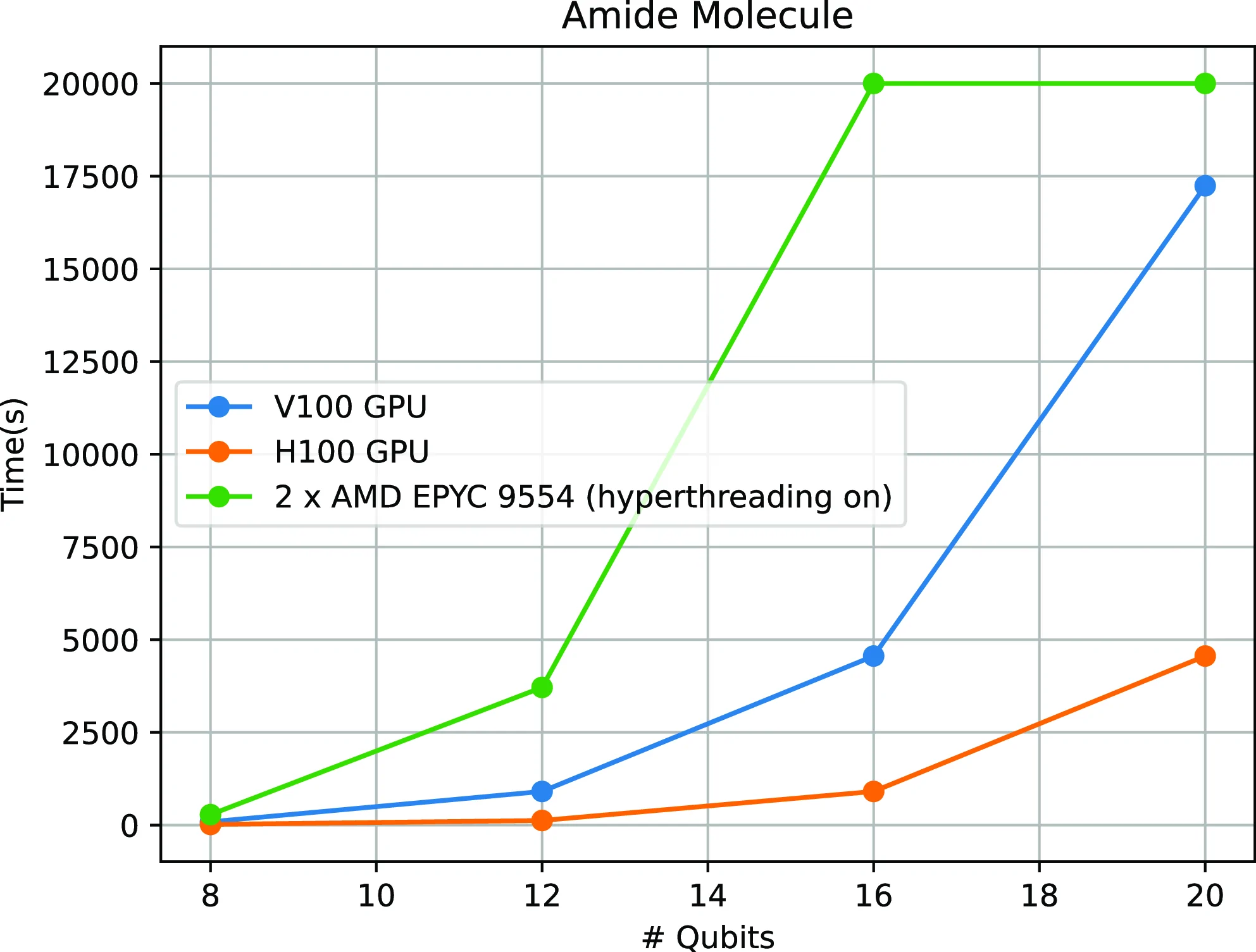
Running times of a VQE cycle on the Amide molecule on GPU V100 and H100 and on AMD EPYC 9554 CPU (hyperthreading on). A time limit of 20,000 s has been imposed. If the value is reported, then the associated VQE did not complete within the time limit.

### 7.5 Identified Challenges

Several limitations constrain the current performance and applicability. The finite-difference force calculation scheme imposes substantial computational overhead, requiring numerous additional VQE optimizations for each gradient evaluation step. While parallelization mitigates this cost, more sophisticated approaches such as analytical gradients^45^ or parameter-shift rules^88^ require future investigation.

The scalability of the finite-difference approach is governed by three coupled factors:

1. The number of atoms (which sets the number of displaced-geometry VQE evaluations to 2 × (3*N*_atoms_ – 6) in internal coordinates, or 2 × 3N_atoms_ in Cartesian coordinates),
2. The cost of each SCF calculation (which provides the prefactor for each displaced-geometry VQE and is mitigated here by warm-starting from the central-geometry solution),
3. The active space size (which dominates the cost of each individual VQE and represents the primary computational bottleneck for the systems studied).

Parallelizing computational effort on displaced geometries could substantially reduce wall-clock time (Section 5.3), but does not change the asymptotic scaling with system size. Switching to analytical gradients would reduce the required VQE count to one per optimization step regardless of molecular size; however, the total time would then be dominated by the cost of evaluating one- and two-electron reduced density matrices from the converged circuit.

Convergence difficulties arise in regions of the potential energy surface with multiple near-degenerate electronic states or when approaching the bond dissociation limits. The variational nature of VQE provides some robustness, but careful initialization and potentially multireference approaches may be necessary for challenging cases.

Although chemically inspired ansätze such as UCCSD offer a stable and consolidated route to systematic accuracy, their rigid structure and rapidly increasing circuit depth limit their applicability in exploratory optimization strategies. In contrast, hardware-efficient ansätze provide a tunable and shallow circuit architecture that supports multiple distinct initializations and parametrizations. This property is crucial for our methodology, which relies on sampling and refining different variational minima through a quasi-adiabatic optimization protocol, an approach that is inherently compatible only with the flexibility of the HEA framework.

The adoption of adaptive ansatze, such as ADAPT-VQE^49,53^ and its gradient-approximated variant GGA-VQE,^89^ represents a natural direction for improving QGO’s wavefunction quality. ADAPT-style procedures dynamically construct the circuit by selecting operators from a chemically motivated pool based on gradient norms, producing expressive circuits whose structures are adapted to the electronic problem at hand. Within the QGO framework, one integration strategy would involve constructing the ADAPT circuit once at the initial geometry, followed by parameter-only relaxation at each subsequent geometry step, analogous to the warm-start adiabatic tracking already employed in the current HEA-based implementation. This would improve the accuracy of both the wavefunction and the computed forces compared to a fixed HEA circuit of comparable depth. The nonadiabatic multistart branching step (Section 4.3) would need to be adjusted to this context, as the multi-initialization logic underlying the current nonadiabatic strategy is specifically tied to the parametric flexibility of HEA; for a fixed ADAPT circuit topology, diversity in local minima exploration would instead rely on parameter-space perturbations within the fixed architecture. GGA-VQE further reduces the quantum overhead associated with operator-pool gradient evaluations, making it particularly attractive for the repeated VQE resolutions required for each geometry step.

Additionally, the choice of the orbital included in the active space is a limiting factor. A small active space is not always able to describe sufficiently a portion of the full correlation of the molecular system, leading to a region of the structure completely untouched by the optimization procedure. Blindly adding more orbitals in the active space has two main drawbacks: it significantly increases the complexity of the requirement to solve each single-point calculation and the orbitals added might not be relevant. A more refined active space selection method ensures that the subspace captures the essential electronic correlations governing molecular structure while remaining computationally tractable on near-term quantum devices. It should be noted that different emerging automated tools for active space selection^90–93^ represent promising alternatives that could further optimize this critical step in future implementations of our methodology.

On the other hand, basis set effects remain important. Our current implementation uses modest basis sets to maintain computational tractability, but achieving chemical accuracy will require larger basis sets and corresponding Pulay corrections.

One of the main challenges that emerged during development was maintaining coherence between the central wavefunction and those reoptimized for infinitesimal displacements. This correspondence in the charge distribution resolved by the self-consistent field (SCF) method was found to be fundamental, since without it, recycling the wavefunction leads to physically meaningless states for the system. More precisely, system’s energy remains unchanged whether the electron cloud—for example, in a π orbital of a benzene fragment—is positively or negatively oriented above or below the plane of symmetry. However, when this distribution is mapped and approximated in a bosonic space such as that of qubits, changes in charge distribution between states cause the wavefunction to vary slightly. Since this is a numerical method, even small variations in the wavefunction-based energy calculation result in large imbalances in the calculated forces.

## 8 Conclusions and Future Work

This work demonstrates the feasibility of molecular geometry optimization using VQE-based approaches at post-HF levels of theory. Our quasi-adiabatic strategy, combined with efficient GPU-accelerated implementations, enables systematic studies of small molecular systems. Benchmark results validate the approach while identifying the areas requiring further development.

We demonstrated that it is possible to *recycle* the central wavefunction across geometry-optimization steps, significantly accelerating computations for pharmaceutically relevant molecular fragments. We demonstrated the feasibility of this hybrid quantum-classical approach for realistic organic molecular fragments, advancing beyond previous literature that tested similar methods only on simple elemental systems such as H_2_, H_3_^+^, H_4_, etc.^20–22^ Our method was natively and purposefully designed to scale efficiently on high-performance-computing (HPC) architectures. The approach is inherently parallelizable, with all quantum circuit components computable via message-passing interface (MPI) paradigms. Specifically, the method offers two levels of parallelization: first, multiple simultaneous searches can be performed to identify optimal starting configurations on the quantum circuit for each molecular geometry; second, gradient calculations for geometry optimization can be executed independently for each Cartesian (or internal) coordinate component of every atom in the molecule.

Long-term prospects include applications to strongly correlated systems, excited-state optimizations, and reaction pathway characterization.^63^ The potential for achieving post-HF accuracy with favorable computational scaling motivates the continued development of these hybrid quantum-classical approaches.

Future work will address current limitations through several directions: implementation of analytical gradient methods^46^ to replace finite-difference force calculations, exploration of adaptive ansatz construction strategies, such as ADAPT-VQE,^53^ CQE,^94^ or (Multi-Threshold) Quantum Information Driven Ansatz,^95–97^ to balance accuracy and circuit depth, extension to larger molecular systems through improved scaling strategies,^54^ and incorporation of quantum error mitigation techniques^98^ for eventual deployment on real quantum hardware.

The integration of quantum computing methodologies into molecular geometry optimization workflows represents a promising avenue for advancing the computational quantum chemistry capabilities. While current implementations remain limited to classical simulations, the framework established here provides a foundation for future developments, as quantum hardware continues to improve.

## Data Availability

The implemented code and tested systems can be found at the linked gitLab page upon request.

## Footnotes

1 depending on the update frequency of the explored minimum-energy landscapes

2 EXSCALATE official Website: www.exscalate.com.

3 DMET: density matrix embedding theory.

4 Transamination is a chemical reaction that transfers an amino group to another molecule.

5 Organic compounds with structure R^1^R^2^C = NR^3^, where R^1/2/3^ are organic groups.
